# Human Herpesvirus 8-Positive Multicentric Castleman Disease in an Immunocompetent Patient: A Diagnostic Challenge

**DOI:** 10.7759/cureus.56534

**Published:** 2024-03-20

**Authors:** Ana Rita G Magalhães, Marta B Santos, Pedro H Almeida, Ana Maria F Carvalho, Beatriz T Exposito

**Affiliations:** 1 Internal Medicine, Centro Hospitalar de Trás-os-Montes e Alto Douro, Chaves, PRT

**Keywords:** immunocompetent, hhv-8, internal medicine in rural areas, clinical profile, multicentric castleman disease

## Abstract

We present a clinical case of a 79-year-old male admitted to inpatient care for longstanding asthenia and respiratory symptoms. Associated features were polyserositis, multiple enlarged lymphatic nodules, acute kidney injury, and heart failure. The patient’s recent medical history revealed SARS-CoV-2 vaccination a week prior and an upper respiratory tract infection. The laboratory results from thoracentesis were compatible with a transudate, with no immunological stain. Epstein-Barr virus polymerase chain reaction (PCR) was positive. The thoracic, abdominal, and pelvic CT scans revealed multiple enlarged lymphatic nodules, worsening the pre-existent polyserositis and hepatosplenomegaly. The patient began to show signs of neurologic symptoms and deterioration of the global health status. An enlarged lymphatic nodule was excised and the pathology showed human herpesvirus 8 multicentric Castleman disease. The disease evolved rapidly into hematological dysfunction and blood transfusions were necessary. Even though the patient was started on high-dose rituximab therapy combined with etoposide, the disease evolved into multiorgan dysfunction with a fatal outcome.

## Introduction

Castleman disease (CD) is a heterogeneous lymphoproliferative disorder that describes a group of disorders that share a spectrum of characteristic histopathological features but have a wide range of poorly understood etiologies, non-specific presentations, different treatments, and outcomes [1,2].

CD was first described in 1954 by Dr. Benjamin Castleman who reported a series of cases with localized mediastinal lymph node enlargement characterized by increased numbers of lymphoid follicles with germinal center involution and marked capillary proliferation, resembling thymomas [1,3]. By the 1980s, CD was divided into unicentric disease (UCD) and multicentric disease (MCD) depending on the location of the affected lymph nodes [1,4]. Human herpesvirus 8 (HHV8) was identified as a potential trigger for the disease in the 1990s and is highly associated with human immunodeficiency virus (HIV) patients [1].

CD can be classified by its regional involvement into UCD when affecting a single lymph node or various lymph nodes circumscribed to one anatomic region and MCD when affecting various lymph nodes in various anatomic regions [3]. The outcome of MCD is frequently negative and rapidly progressive.

We present the case of a 79-year-old immunocompetent male admitted with non-specific longstanding symptoms, with the histological diagnosis of HHV8-positive MCD.

The non-specific symptoms and the lymph node enlargement, which are the hallmarks of the disease, can mimic many other malignant or non-malignant entities such as lymphoproliferative diseases, thus delaying the diagnosis [4,5].

This clinical case was presented at the 27th National Congress of Internal Medicine in Vila Moura, Portugal on October 6th, 2021.

## Case presentation

A 79-year-old autonomous man presented to the emergency room (ER) with longstanding asthenia, cough, night sweats, and unintentional weight loss. He also reported orthopnea. His medical history was relevant for hypertension, chronic hypertensive renal disease, hyperlipidemia, chronic gastritis, and iron deficiency anemia. His recent medical history revealed an upper respiratory tract infection, for which outpatient treatment was done, and SARS-CoV-2 vaccination a week prior. There was a record of moderate alcohol consumption. There was no history of tobacco or recreational drug usage.

**Figure 1:**
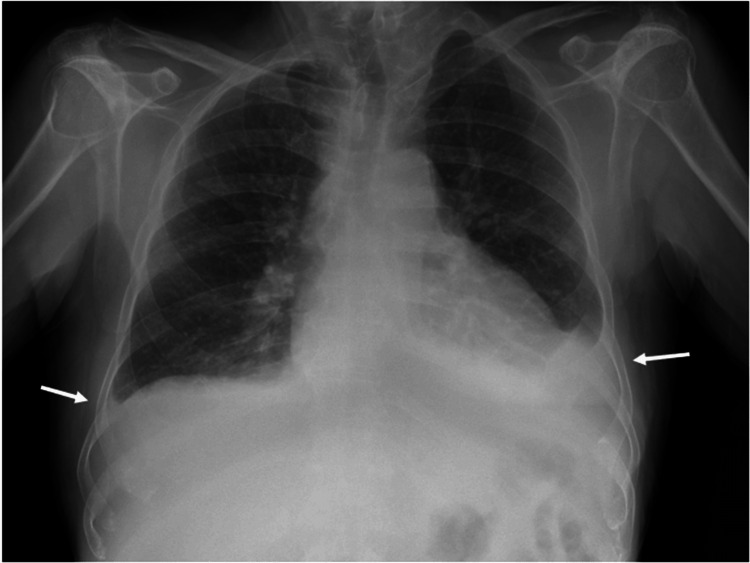
Bilateral pleural effusion visible on the thoracic X-ray. The arrows point to the pleural effusion.

In the ER, the thoracic X-ray suggested a slight bilateral pleural effusion (Figure 1), alongside the laboratory results that denoted a slight worsening of renal function (Table 1). Thus, decompensated heart failure was assumed as the working diagnosis, and the patient was admitted for inpatient care, treatment, and investigation.

**Table 1:** Laboratory results and evolution. RBC = red blood cells; WBC = white blood cells; CRP = C=reactive protein; IgA = immunoglobulin A; IgG = immunoglobulin G;  IgM = immunoglobulin M

Parameter	Normal range	Upon admission	Evolution
RBC	4.4–6.0 × 10^6^/µL	4.09 × 10^6^/µL	2.57 × 10^6^/µL
Hemoglobin	13–18 g/dL	11.40 g/dL	7.20 g/dL
WBC	4.0–11 × 10^3^/µL	5.52 × 10^3^/µL	4.86 × 10^3^/µL
Neutrophiles	53.8–69.8%	53.9%	56%
Lymphocytes	25.3–47.3%	19.2%	22.7%
Platelets	150–400 × 10^3^/µL	211 × 10^3^/µL	66 × 10^3^/µL
Serum creatinine	0.7–1.4 mg/dL	1.6 mg/dL	2.10 mg/dL
Serum urea	<50 mg/dL	105 mg/dL	140 mg/dL
Glomerular filtration rate	100–130 mL/minute/1.73m^2^	41.88 mL/minute/1.73 m^2^	30.60 mL/minute/1.73 m^2^
CPR	<0.5 mg/dL	8.49 mg/dL	9.60 mg/dL
D-dimer	0–0.5 µg/mL	3.8 µg/dL	-
Serum IgG	60–1,560 mg/dL	1,680 mg/dL	-
Serum IgA	90–410 mg/dL	427 mg/dL	-
Serum IgM	30–360 mg/dL	56 mg/dL	-

The patient developed a fever on the third day of admission, and his general health condition further declined. Lab results showed high D-dimers, polyclonal gamma spikes in the serum protein electrophoresis, and elevated titers of beta-2 microglobulin. Detailed evolution of laboratory parameters can be seen in Table 1. The bacteriological study was negative, as well as autoimmunity. The virus panel revealed a positive polymerase chain reaction (PCR) for the Epstein-Barr virus (Table 2). At this point, the differential diagnosis included B-cell lymphoma or POEMS syndrome.

**Table 2:** Laboratory results for infectious agents, tumor biomarkers, and autoimmunity. *: anti-cardiolipin antibody: negative <10, undetermined 10-40, positive >41. ANA = antinuclear antibodies; ANCA = antineutrophil cytoplasmic antibodies; anti-CCP = anti-cyclic citrullinated peptide; anti-MPO = anti-myeloperoxidase; BK virus = human polyomavirus 1; CA-19.9 = carbohydrate antigen 19.9; CEA = carcinoembryonic antigen; CMV = cytomegalovirus; C3c = complement system protein C3c; C4 = complement system protein C4; EBV = Epstein-Barr virus; HBs = hepatitis B s protein; HCV = hepatitis C virus; HIV = human immunodeficiency virus; PSA = prostate-specific antibody; - = normal range not available on lab results

Viral serology	Result	Normal range
CMV IgG	575.3	<6.0
CMV IgM	0.200	<0.7
HBs antigen	0.43 – non-reactive	-
HBs antibody	84.02 - reactive	-
HCV antibody	0.09 - non-reactive	-
HIV 1 and 2 antibody	0.24 - non-reactive	-
Paul Bunnel	Negative	-
EBV DNA	Positive	-
Syphilis antibodies	Negative	-
BK virus DNA	Negative	-
Tumor biomarkers		
CEA	1.7 ng/mL	0.0–3.0
CA-19.9	5 U/mL	0–37
Alpha-fetoprotein	0.7 IU/mL	1–8
Beta-2 microglobulin	9.38 µg/mL	0.8–2.2
PSA	0.200 ng/mL	<0.9
Autoimmunity		
ANA	<1:160	<1:160
ANCA	<1:20	<1:20
C3c	119	90 - 180
C4	15	12 - 36
Anti-cardiolipin antibody IgG	15.0	Negative <10*
Beta 2 glicoprotein IgG	5.8	<7.0
Anti-CCP antibody	2.7	<7.0
Anti-SSA antibody	2.0	<7.0
Anti-SSB antibody	1.1	<7.0
Anti-Sm antibody	2.5	<7.0
Anti-MPO antibody	2.9	<3.5
Anti-thyroglobulin antibody	21	<40

**Figure 2:**
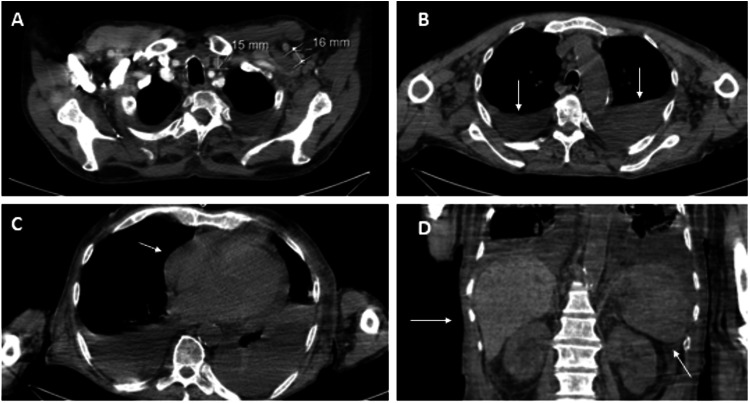
Findings on CT scan. A: Multiregional lymph nodes over 1 cm in diameter. B: Pleural effusion. C: Pericardial effusion. D: Hepatomegaly and splenomegaly.

**Figure 3:**
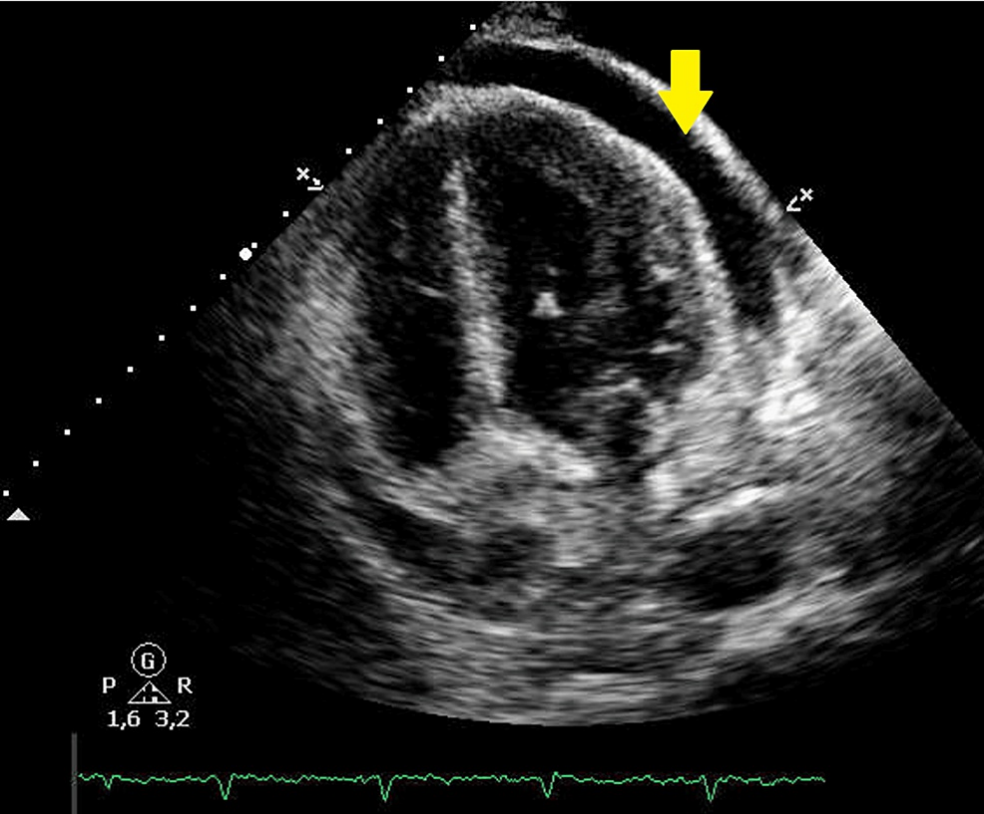
Pericardial effusion on echocardiogram. The arrow points to the large pericardial effusion visible with this imaging method.

Thoracic, abdominal, and pelvic CT scans showed polyserositis (predominantly pleural (see Figure 1 and Figure 2B) and pericardial effusion (see Figure 2C and Figure 3)), multiple and dispersed enlarged lymph nodes (the largest being 18 mm), and hepatosplenomegaly (see Figure 2A and Figure 2D, respectively).

First, a diagnostic thoracentesis was performed. The pleuritic effusion was clear and citric in color, and its labs were compatible with a transudate, with predominant mononuclear cells upon observation. No abnormal cells were found in this liquid, and cultures were negative. An excisional biopsy of an enlarged axillary lymph node was done. The microscopic analysis showed a hyperplastic ganglion containing plasma cells and a vascular hyaline appearance. Immunophenotyping of the lymph node was positive for HHV8. A diagnosis of HHV8-positive MCD was made.

The patient was allocated to hematology inpatient care, and combination therapy with methylprednisolone, rituximab, and etoposide was started. Despite the efforts and intensive therapy, the disease rapidly evolved into systemic multiorgan dysfunction and resulted in death.

## Discussion

CD is a heterogeneous lymphoproliferative disorder that encompasses at least four different diseases sharing a spectrum of characteristic histopathological features [1]. Therefore, there is a wide range of poorly understood etiologies, presentations, and treatments. The disease can be classified according to regional involvement as UCD and MCD. The most common characteristics of both UCD and MCD are displayed in Table 3 [2,3].

**Table 3:** Classification of Castleman disease according to regional involvement.

Unicentric Castleman disease	Multicentric Castleman disease
≥1 involved lymphatic ganglia circumscribed to one region	≥1 involved lymphatic ganglia in more than one region
The most common type	Usually presents with systemic symptoms
Any age	Occurs more often in males
Slightly more common in females	More common after 60 years old
Usually with no systemic symptoms	
Exaggerated organic reactions to antigens or neoplastic process	

UCD involves lymph nodes of a single region and usually does not cause systemic symptoms. On the other hand, MCD tends to cause systemic symptoms and involves multiple lymphatic nodules in different regions. The latter can be idiopathic (iMCD, iMCD associated with TAFRO syndrome), HHV8 associated, and POEMS associated [1,3,4,6-8] (Table 4).

**Table 4:** Characteristics of the MCD spectrum diseases. iMCD = idiopathic multicentric Castleman disease; iMCD-TRAFO = idiopathic multicentric Castleman disease associated with TAFRO syndrome (TAFRO syndrome is a condition characterized by thrombocytopenia, anasarca, fever, reticulin myelofibrosis (or renal insufficiency), and organomegaly (hepatosplenomegaly and lymphadenopathy)) [6]; POEMS MCD = multicentric Castleman disease associated with POEMS (polyneuropathy, organomegaly, endocrinopathy, monoclonal plasma cell disorder, skin changes) [7]; HHV8 MCD = multicentric Castleman disease associated to human herpesvirus 8 infection; +/- = can be either present or absent; ++ = often present; +++ = very often present; N/A = not analyzed

	iMCD	iMCD-TAFRO	POEMS MCD	HHV8 MCD
Age	50–69 years	50–59 years	50–59 years	50–59 years
Systemic symptoms	++	+++	++	+++
Organomegaly	++	+++	+++	+++
Anemia, thrombocytopenia	++ (Predominantly thrombocytosis)	+++	+/-	+++
Other characteristics	Hypergamma-globulinemia	Anasarca	N/A	HIV + Kaposi hypergamma-globulinemia
Prevalent histopathology	Plasmacytic	Mixed/hypervascular	Mixed/plasmacytic	Plasmacytic
Clinical course	Variable	Very aggressive	Aggressive	Aggressive
First-line therapy	IL-6-targeted therapy; rituximab; systemic therapies	IL-6-targeted therapy; rituximab; systemic therapies; calcineurin inhibitors	Radiation; Myeloma-type therapy	Rituximab; etoposide

The different diseases can also be grouped based on histopathologic subtypes as hypervascular, plasmocytic-rich, or mixed-type disease [1,8]. The hypervascular subtype is more common in UCD, and it preserves the overall ganglia architecture. The plasmocytic-rich subtype occurs predominantly in HHV8-associated multicentric disease, iMCD, and MCD POEMS associated. The mixed type combines the histological characteristics of the other subtypes.

MCD spectrum diseases occur with flares of non-specific symptoms, suggesting an underlying chronic inflammatory syndrome [4].

HHV8-associated MCD is responsible for nearly 50% of MCD cases, with immunodeficiency being the primary risk factor for developing the disease [5]. HIV infection is the most common underlying immunocompromised state [1,5]. Only a minority of HHV8-infected individuals develop HHV8-MCD, and the period between the primary infection and the disease can range from years to decades, emphasizing the role of triggers in determining the evolution of such diseases.

Some conceptual frameworks of HHV8-MCD pathogenesis suggest that the clinical manifestations can be reactive changes to elevated levels of interleukin 6 (IL-6) and other circulating factors in the cytokine and chemokine storm [5]. The viral infection and activation can increase the production of viral IL-6, which shares a 25% similarity with human IL-6 [3]. This cytokine is multifunctional, inducing the differentiation and proliferation of B and T cells. It is also responsible for promoting the synthesis of acute-phase proteins that may lead to systemic symptoms [5].

HHV8-MCD is usually aggressive in its clinical course. The mainstay therapies include rituximab and etoposide-based therapies. Rituximab can be used as a single-agent therapy, administered weekly for four weeks in patients with adequate performance status and absence of hemophagocytic syndrome, hemolytic anemia, or end-organ damage [1]. Etoposide is added to the rituximab-based therapy in high-risk patients.

## Conclusions

HHV8-positive MCD occurs predominantly in immunocompromised patients, usually linked to HIV, and typically has a fast and aggressive progression, sometimes mimicking malignant diseases such as B-cell lymphoma. Nonetheless, it can also occur in immunocompetent patients, as illustrated by this clinical case. As the prognosis is often poor, and the progression is rather fast, clinicians must consider this entity even in immunocompetent patients. Failure to detect serious illnesses or deterioration, particularly due to healthcare complexity, can be detrimental.
